# The Biphasic Root Growth Response to Abscisic Acid in Arabidopsis Involves Interaction with Ethylene and Auxin Signalling Pathways

**DOI:** 10.3389/fpls.2017.01493

**Published:** 2017-08-25

**Authors:** Xiaoqing Li, Lin Chen, Brian G. Forde, William J. Davies

**Affiliations:** Lancaster Environment Centre, Lancaster University Lancaster, United Kingdom

**Keywords:** abscisic acid (ABA), Arabidopsis, auxin transport, auxin signalling, ethylene biosynthesis, ethylene signalling, hormone, root elongation

## Abstract

Exogenous abscisic acid (ABA) is known to either stimulate or inhibit root growth, depending on its concentration. In this study, the roles of ethylene and auxin in this biphasic effect of ABA on root elongation were investigated using chemical inhibitors and mutants. Inhibitors of ethylene perception and biosynthesis and an auxin influx inhibitor were all found to block the inhibitory effect of high ABA concentrations, but not the stimulatory effect of low ABA concentrations. In addition, three ethylene-insensitive mutants (*etr1-1*, *ein2-1*, and *ein3-1*), two auxin influx mutants (*aux1-7*, *aux1-T*) and an auxin-insensitive mutant (*iaa7/axr2-1*) were all insensitive to the inhibitory effect of high ABA concentrations. In the case of the stimulatory effect of low ABA concentrations, it was blocked by two different auxin efflux inhibitors and was less pronounced in an auxin efflux mutant (*pin2/eir1-1*) and in the *iaa7/axr2-1* auxin-insensitive mutant. Thus it appears that the stimulatory effect seen at low ABA concentrations is *via* an ethylene-independent pathway requiring auxin signalling and auxin efflux through PIN2/EIR1, while the inhibitory effect at high ABA concentrations is *via* an ethylene-dependent pathway requiring auxin signalling and auxin influx through AUX1.

## Introduction

Plant growth and yield production are often limited by a variety of abiotic stresses in agricultural systems (Cramer et al., 2011). A root system that is able to efficiently take up water and nutrient from the soil is crucial for plant growth and functioning, particularly if the plant is to accumulate any yield when water and nutrients are in short supply (Hammer et al., 2009; Hodge et al., 2009; Hodge, 2010). Previous studies have reported that mild soil drying stimulates root growth, but when soil drying becomes more severe, it inhibits root growth (Sharp and Davies, 1979; Watts et al., 1981). However, there is no consensus on the mechanisms underlying these root responses. An improved understanding of the mechanistic basis of root growth and development will be useful both for novel crop management and breeding aimed at plant improvement to at least maintain yield in different environments under a changing climate.

Plant hormones are crucial regulators of plant growth and development (Davies, 2010). Among them, abscisic acid (ABA) has been recognised as a stress-hormone and its regulation of plant drought responses has been extensively studied (e.g., Hsiao, 1973; Schachtman and Goodger, 2008; Cutler et al., 2010). ABA accumulates under soil drying and the endogenous ABA concentration in the plant can be an indicator of soil water availability (Zhang and Davies, 1989). Generally, ABA is known as an inhibitor of shoot and root growth of plants under well-watered conditions (Sharp et al., 1994; Sharp and LeNoble, 2002) and previous studies have shown that ABA acts as an inhibitor of growth of plants under water deficit (Bensen et al., 1988; Creelman et al., 1990; Rowe et al., 2016). On the other hand, maize plants with reduced endogenous ABA content (by genetic modification or chemical treatments) had roots that were more sensitive to the inhibitory effect of low water potential, indicating that ABA plays a role in maintaining root elongation under low water potentials (Saab et al., 1990). However, other studies have indicated complex biphasic effects of exogenous ABA on root growth under well-watered conditions, where relatively low concentrations of ABA stimulated root growth while high concentrations inhibited root growth (Watts et al., 1981; Xu et al., 2013). This is analogous to the biphasic effects of soil drying on root growth where mild water deficit stimulated root growth while more severe water deficit inhibited root growth (Sharp and Davies, 1979; Watts et al., 1981; Creelman et al., 1990).

Ethylene is another major hormone that mediates plant responses to abiotic stresses, including water deficit (Morgan and Drew, 1997; Spollen et al., 2000; Davies, 2010). Ethylene and its precursor 1-aminocyclopropane-1-carboxylic acid (ACC) have been reported to inhibit root cell elongation, thus inhibiting root growth (Le et al., 2001; Růžička et al., 2007). Alarcón et al. (2009) found that root elongation rate in maize decreased as ethylene production increased. The involvement of ethylene in ABA-regulated root growth was investigated in further detail by Beaudoin et al. (2000) and Ghassemian et al. (2000) who found that root growth in a number of ethylene signalling mutants was less sensitive to ABA (1–150 μM) which caused inhibition on root growth, but that the ethylene biosynthesis inhibitor aminoethoxyvinylglycine (AVG) even enhanced sensitivity to the inhibition of root growth caused by ABA (Ghassemian et al., 2000). These results indicated ethylene signalling plays a positive role in inhibiting root growth under high ABA concentration, but this inhibition does not necessarily involve *de novo* ethylene biosynthesis. In partial contradiction, a recent study found that ethylene biosynthesis is necessary for the inhibitory effect of high ABA concentration on root growth (Luo et al., 2014). To our knowledge, a role for ethylene in the stimulatory effect of low ABA concentrations on root growth has not been explored.

The hormone auxin is generally recognised as a master regulator in plant root development (Saini et al., 2013). Studies using mutants and protein analysis have provided evidence for crosstalk between auxin and ABA signalling pathways in the root (Bianchi et al., 2002; Rock and Sun, 2005). Mutants that are resistant to both auxin and ABA (e.g., *axr2*) also provided genetic evidence for the interaction between auxin and ABA signalling pathways (Pickett et al., 1990; Wilson et al., 1990; Tian and Reed, 1999). However, the external ABA concentrations applied in these earlier studies were relatively high (1–150 μM), based on previous estimates of the ABA concentration in well-watered Arabidopsis root tips as 100 ng g^-1^ FW (Xu et al., 2013) (corresponding to a tissue concentration of approximately 0.5 μM). Lower ABA concentrations (0.1 μM) were used in a recent study which reported a role for ABA in modulating auxin transport in the Arabidopsis root apex to maintain root growth under moderate water stress (Xu et al., 2013). However, the role of the auxin signalling pathway in the response to low ABA concentrations has not yet been examined. In addition, how auxin transport could involve in root responses to ABA, especially high ABA concentrations, is less clear.

Tissue ABA concentrations can gradually increase to more than 30 times that in the well-watered plants as soil water content slowly decreases (Zhang and Davies, 1989), or up to 10 times that in non-stressed plants when plants were subjected to salt stress (up to 300 mM NaCl) (Jia et al., 2002). Therefore, investigating the effects of external application of both low and high concentrations of ABA on plant root growth, and the involvement of other hormones, i.e., auxin and ethylene, can improve our understanding of how plants respond to different levels of stress (e.g., water stress). Such understanding may then facilitate research aimed at enhancing plant performance under those abiotic stresses. Here, we hypothesise that the biphasic effect of low and high ABA concentrations on root growth involves auxin and ethylene. Five chemical inhibitors and 12 mutant lines that are relevant to ethylene and auxin signalling were used to test this hypothesis.

## Materials and Methods

### Plant Materials

The wild-type accession of *Arabidopsis thaliana* L. used in this study was Col-8 (European Arabidopsis Stock Centre catalogue no. N60000). Besides, the auxin influx *AUX1* mutants *aux1-T* (N657534), *aux1-7* (N9583); the auxin efflux mutants *pin2/eir1-1* (N8058), *pin3-4* (N9363), *pin3-5* (N9364), *pin4-3* (N9368), and *pin7-2* (N9366); and auxin signalling mutants *iaa7/axr2-1* (N3077) and *tir1-1* (N3798) were obtained from the European Arabidopsis Stock Centre. The ethylene-insensitive mutants *etr1-1* (*ETHYLENE RESPONSE 1*) (Bleecker et al., 1988), *ein2-1* (*ETHYLENE INSENSITIVE 2*) (Guzmán and Ecker, 1990), and *ein3-1* (Roman et al., 1995) were kindly provided by Dr. Mike Roberts (Lancaster University, United Kingdom). The auxin reporter line *DR5::GFP* (Ottenschläger et al., 2003) was a kind gift from Prof. Klaus Palme (University of Freiburg, Germany). All Arabidopsis lines were in the Columbia background.

Surface-sterilised seeds were sown on solid medium containing 0.02 x B5 medium, 1 mM KNO_3_, 0.5% (w/v) sucrose and 1% agar in 90 mm diameter Petri dishes (Zhang and Forde, 1998). After stratifying the seed in the dark (4°C) for 2–3 days, the Petri dishes were incubated in a vertical orientation in a growth room at 22°C with a 16 h light period and an irradiance of 100 μmol m^-2^ s^-1^. Four to five days later, seedlings with similar root length were transferred to fresh plates containing ABA at different concentrations. Five inhibitors were added to the growth medium as required: namely, the ethylene biosynthesis inhibitor AVG (0.3 or 0.5 μM) (A6685, Sigma-Aldrich); the ethylene perception inhibitor silver thiosulfate (STS, 10 μM); and the auxin efflux inhibitors *N*-1-naphthylphthalamidic acid (NPA, 10 μM) (PS343, Sigma-Aldrich), 2,3,5-triiodobenzoic acid (TIBA, 10 μM) (T5910, Sigma-Aldrich); and the auxin influx inhibitor 3-chloro-4-hydroxyphenylacetic acid (CHPAA, 10 μM) (224529, Sigma-Aldrich). For plates with treatments, 3–6 seedlings were placed on 9 cm diameter plates (25 ml medium), or 7–9 seedlings on 12 cm square plates (50 ml medium). The top one-fifth of the agar medium was excised so that the shoot was not in direct contact with the medium. ABA (A1296, Sigma-Aldrich) stock solutions were made in 10 mM (+ABA) with 0.03 M KOH. A 60 mM STS solution was freshly prepared by mixing 300 mM silver nitrate with 300 mM sodium thiosulphate in a 1:4 (v/v) ratio.

### Root Growth Measurements

Primary root growth was monitored during the 3–6 days after seedlings were transferred to the treatment plates by marking the position of the root tips on the base of the plate at 24 or 48 h intervals. At the end of each experiment, the plates were imaged on a flat-bed scanner with a ruler as the reference. The images were semi-manually analysed using Optimas Image Analysis software (Version 6.1 Media Cybernetics, Inc., United States) for root length. The primary root lengths at the beginning and the end of an experiment were presented in Supplementary Table 1.

### Confocal Microscopy

After 3 days of ABA treatments, *DR5::GFP* seedlings were stained briefly (50 s) with 10 μM propidium iodide. GFP and propidium iodide fluorescence was then detected using a Leica SP2-AOBS confocal laser scanning microscope and the images were electronically superimposed using LCS Lite software (Leica, Germany). Quantification of the GFP fluorescence signal was performed using ImageJ (National Institutes of Health, United States).

### Statistical Analysis

The statistical software SPSS 21.0 (IBM, United States) was used to perform one-way or two-way ANOVA with Tukey’s *post hoc* test at the *P* < 0.05 level. The effect size of those ANOVA was reported by eta^2^ or partial eta^2^. The criteria for effect size: no effect, eta^2^ = 0; small, eta^2^ = 0.0099; medium, eta^2^ = 0.0588; large, eta^2^ = 0.1379 (Richardson, 2011).

## Results

### Effect of Exogenous ABA on Root Growth

**FIGURE 1:**
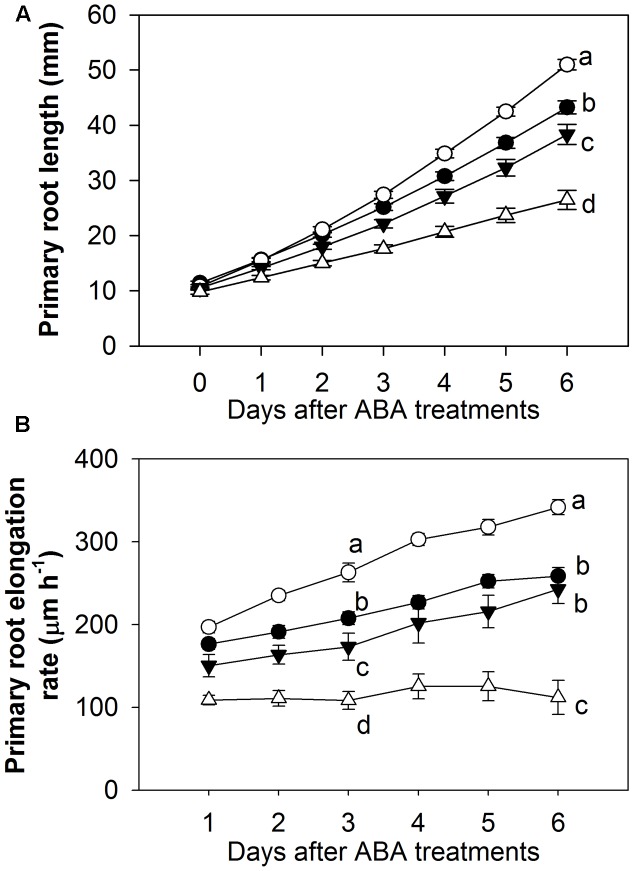
Biphasic effect of applied exogenous ABA on the growth of primary root over the 6-day treatments. **(A)** Total primary root length. **(B)** Primary root elongation rate. Four-day old Arabidopsis wild-type Col-8 seedlings with similar root length were chosen and transferred to newly made 0.02 × B5 medium (1 mM KNO_3_, 0.5% sucrose) with various ABA concentrations (black circle, control; white circle, 0.1 μM ABA; black triangle, 1 μM ABA; white triangle, 10 μM ABA). Primary root length was marked after transplanting and the increase of primary root were measured every day. The root elongation rate was calculated for each day. The values are means, and the vertical bars represent standard errors. Data analysed using one-way ANOVA with Tukey’s *post hoc* test and different letters indicate significant differences among ABA treatments in the same day at *P* < 0.05. Eta^2^ of one-way ANOVA: **(A)** 0.776 (day 6); **(B)** 0.694 (day 3); 0.737 (day 6). Seedling numbers: control, *n* = 14; 0.1 μM ABA, *n* = 9–14; 1 μM ABA, *n* = 10–14; 10 μM ABA, *n* = 11–14. At least three independent experiments were performed and similar results obtained and reported.

A detailed comparison of the effects of a range of ABA concentrations on root elongation was performed by transferring 4 day-old Arabidopsis seedlings to vertical agar plates containing 0 (control), 0.1, 1, and 10 μM ABA and measuring the increase in root length at daily intervals over the following 6 days (**Figure 1**). The results showed that 10 μM ABA inhibited root growth by about 40% while 0.1 μM ABA stimulated growth by almost 20% when measured over the 6-day period (**Figure 1A**). The stimulatory effect of 0.1 μM ABA persisted over the duration of the treatment and by the 6th day the roots were growing at a rate which was more than 30% faster than the control (**Figure 1B**). It appears that the intermediate concentration of ABA used (1 μM) is close to the threshold for the transition from stimulation to inhibition as it had little effect on root elongation (**Figures 1A,B**). In subsequent experiments, concentrations less than 1 μM ABA (usually 0.1 μM ABA) were therefore used for studying the stimulatory effect of low ABA concentrations and concentrations greater than 1 μM ABA (usually 10 μM ABA) were used for studying the inhibitory effect of high ABA concentrations.

### Investigating the Role of Ethylene in the Root Responses to High and Low Concentrations of ABA

**FIGURE 2:**
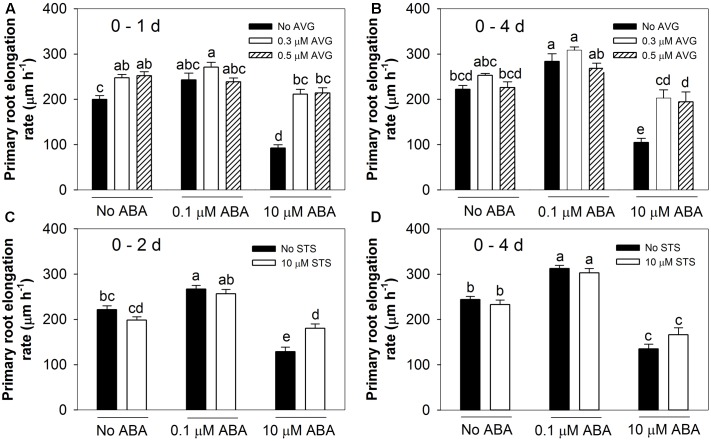
Ethylene biosynthesis and signalling inhibitors altered root responses to ABA treatments. AVG: ethylene biosynthesis inhibitor. STS: ethylene signalling inhibitor. **(A)** The effects of AVG after 1 day. **(B)** The effects of AVG over a 4-day period. **(C)** The effects of STS during the first 2-day period. **(D)** The effects of STS during 4 days. Col-8 seedlings were germinated, chosen and transferred to medium as described in **Figure 1**. The medium was treated with various ABA and AVG/STS concentrations (μM). Primary root length was marked after transplanting and the increase of primary root were measured every day. The root elongation rate was calculated for the 1 or 2 days and 4 days after treatments on average. The values are means, and the vertical bars represent standard errors of the means. Data analysed using one-way ANOVA with Tukey’s *post hoc* test and different letters indicate significant differences cross all treatments at *P* < 0.05. Eta^2^: **(A)** 0.650; **(B)** 0.622; **(C)** 0.729; **(D)** 0.817. Seedling numbers: **(A)** *n* = 14; **(B)** *n* = 9–14; **(C)** *n* = 9–12; **(D)** *n* = 7–12. At least three independent experiments were performed and similar results obtained and reported.

It has previously been established that the inhibitory effect of high ABA concentrations on root growth is an ethylene-dependent process (Ghassemian et al., 2000). To confirm these findings under our experimental conditions and to investigate whether the stimulatory effect of low ABA concentrations is also ethylene-dependent, seedlings were treated with different concentrations of ABA in the presence or absence of either AVG (an ethylene biosynthesis inhibitor) or STS (an ethylene perception inhibitor). The primary root elongation rates were determined over a 4-day period of treatment. When 0.3 or 0.5 μM AVG was included along with the 10 μM ABA treatment, the inhibitory effect was relieved as measured after either 1 day (**Figure 2A**) or 4 days (**Figure 2B**).

In contrast to AVG’s ability to interfere with the inhibitory effect of ABA, the presence of either 0.3 or 0.5 μM AVG had no influence on the trend of the stimulatory effect after adding 0.1 μM ABA (**Figures 2A,B**). In addition, AVG treatment tended to promote root growth which might mask the stimulatory effect of ABA at low concentrations (**Figures 2A,B** and data not shown). Thus while ethylene biosynthesis is required for the inhibitory effect of high ABA concentrations it is not required for the stimulatory effect of low ABA concentrations.

When 10 μM STS was used to interfere with ethylene perception it almost completely overcame the inhibitory effect of 10 μM ABA when measured after the first 2 days of treatment (**Figure 2C**). This antagonistic effect was lost when root growth was measured over a 4-day period (**Figure 2D**), which we attribute to the known instability of STS (Ag^+^) when exposed to light. However, when included along with 0.1 μM ABA, the STS did not interfere with the stimulatory effect on root growth as measured after either 2 or 4 days (**Figures 2C,D**). Therefore, the inhibitory effect of high ABA concentrations, but not the stimulatory effect of low ABA concentrations, could be eliminated by interfering with ethylene perception.

**FIGURE 3:**
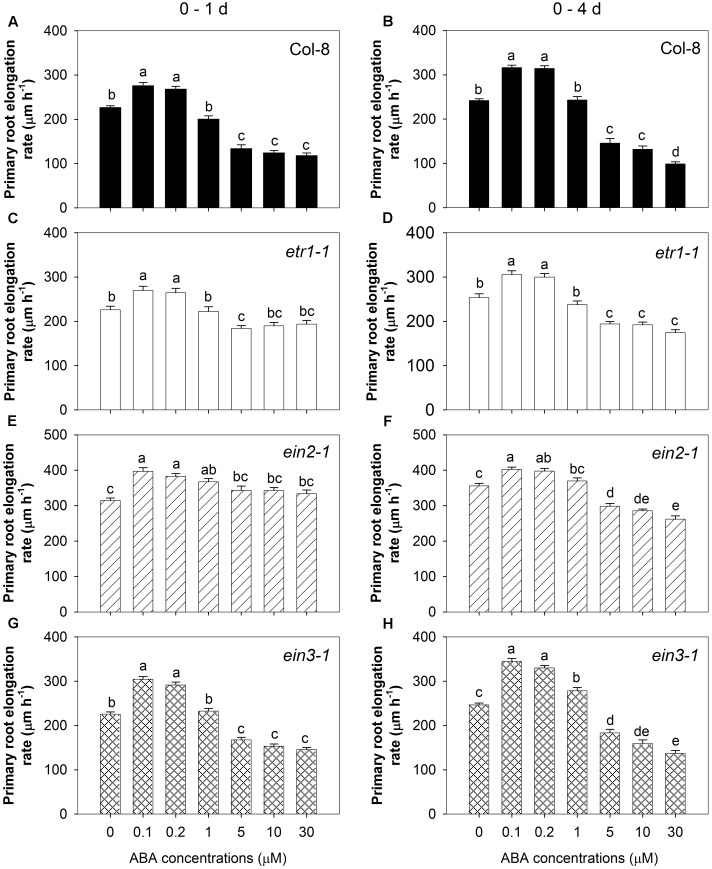
Various responses of root growth to seven ABA treatments in three ethylene insensitive mutants and wild-type. Primary root elongation rates 1 day after treatment: **(A)** Col-8 wild-type; **(C)** *etr1-1*; **(E)** *ein2-1*; **(G)** *ein3-1*, and over a 4-day treatment: **(B)** Col-8 wild-type; **(D)** *etr1-1*; **(F)** *ein2-1*; **(H)** *ein3-1*. Seedlings of each line were germinated, chosen and transferred to medium with various ABA concentrations (μM) as described in **Figure 1**. Primary root length was marked after transplanting and the increase of primary root were measured every day. The root elongation rate was calculated for 1 and 4 days after treatments on average. Only one genotype was used in each experiment (*n* = 14), and results for each genotype came from combining two sets of independent experiments. All eight experiments were done consecutively from 17/07/2013 (day/month/year) to 26/08/2013. The values are means, and the vertical bars represent standard errors of the means. Data analysed using one-way ANOVA with Tukey’s *post hoc* test and different letters indicate significant differences among ABA treatments at *P* < 0.05. Eta^2^: **(A)** 0.773; **(B)** 0.842; **(C)** 0.333; **(D)** 0.635; **(E)** 0.237; **(F)** 0.660; **(G)** 0.810; **(H)** 0.827. Seedling numbers: **(A)** *n* = 28; **(B)** *n* = 21–28; **(C)** *n* = 28; **(D)** *n* = 22–28; **(E)** *n* = 28; **(F)** *n* = 21–28; **(G)** *n* = 28; **(H)** *n* = 27–28. At least three independent experiments were performed and similar results obtained and reported.

To look further into the role of ethylene signalling in the two components of the root response to ABA, seedlings of three ethylene-insensitive mutants (*etr1-1*, *ein2-1*, and *ein3-1*) were treated with a range of concentrations of ABA. Two-way ANOVA showed that the primary root elongation rate was affected by genotype, ABA treatment and their interaction in the first 24 h and 4 days after treatment (*P* < 0.0001, Supplementary Table 2). Thus, the four genotypes responded to those ABA treatments differently and the effect of those two factors depend on each other. **Figure 3** displays the effect of ABA treatment in each genotype, while Supplementary Figure 1 shows the effect of genotype under each ABA treatment.

All three mutants to varying degrees showed a diminished response to the inhibitory effect of high [ABA] compared to the wild-type (**Figure 3**). This was particularly evident in *etr1-1* and *ein2-1* during the 1st day of treatment when even the highest concentration of ABA (30 μM) had no effect on the root elongation rate, and inhibited root elongation by only 14% in *etr1-1* and even stimulated root elongation by 6% in *ein2-1* compared to 48% inhibition in the wild-type (**Figures 3A,C,E**). A much less pronounced effect was seen in *ein3-1*, where 30 μM ABA inhibited root elongation by 35% over the 1st day of treatment (**Figure 3G**). Over the 4-day period of treatment the inhibitory effect of the high ABA concentrations was stronger in all lines, but the same pattern of decreased sensitivity in the mutants was observed (**Figures 3B,D,F,H**). The low ABA concentrations (0.1 and 0.2 μM) stimulated root elongation of the wild-type by ∼20% in the 1st day after treatment and by ∼30% over the full 4 days of treatment (**Figures 3A,B**). Similarly, the low ABA concentrations also stimulated root elongation of the three ethylene-insensitive mutants as seen after either 1 or 4 days (**Figures 3C–H**). These results confirmed the evidence from the STS treatment (**Figures 2C,D**) that ethylene signalling is important for the inhibitory effect of high [ABA], but not for the stimulatory effect of low [ABA].

### Investigating the Role of Auxin Transport and Signalling in the Root Responses to ABA

**FIGURE 4:**
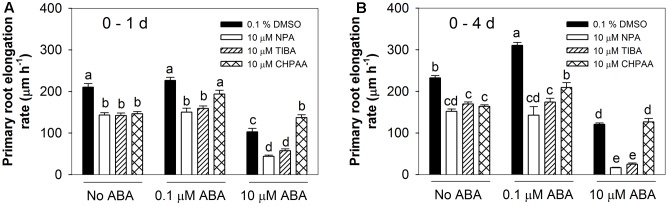
Auxin influx and efflux inhibitors altered root responses to ABA. NPA, *N*-1-naphthylphthalamidic acid, auxin efflux inhibitor; TIBA, 2,3,5-triiodobenzoic acid, auxin efflux inhibitor; CHPAA, 3-chloro-4-hydroxyphenylacetic acid, auxin influx inhibitor. Primary root elongation rates **(A)** 1 day after treatment and **(B)** over a 4-day treatment period. Col-8 seedlings were germinated, chosen and transferred to medium as described in **Figure 1**. The medium was treated with various ABA concentrations and 0.1% DMSO or 10 μM NPA/TIBA/CHPAA. Primary root length was marked after transplanting and the increase of primary root were measured every day. The root elongation rate was calculated for 1 day and over a 4-day treatment on average. The values are means, and the vertical bars represent standard errors of the means. Data analysed using one-way ANOVA with Tukey’s *post hoc* test and different letters indicate significant differences cross all treatments at *P* < 0.05. Eta^2^: **(A)** 0.834; **(B)** 0.951. Seedling numbers: **(A)** *n* = 10–12; **(B)** *n* = 3–12. At least three independent experiments were performed and similar results obtained and reported.

To investigate the role of auxin transport in the root responses to ABA, two auxin efflux inhibitors (NPA and TIBA) and an auxin influx inhibitor (CHPAA) were firstly employed in this study. In this experiment, the stimulatory effect of the low ABA concentration (0.1 μM) was only seen after 4 days treatment and not after the 1st day (**Figure 4**). However, some other repeated experiments showed clear effect of low ABA concentration (data not shown). This discrepancy during the first 24 h may be due to changes of new batches of chemicals during this study and some other unknown factors. When seedlings were grown for 4 days in the presence of either of the auxin efflux inhibitors, the stimulatory effect of 0.1 μM ABA was no longer observed (**Figure 4B**). However, in the presence of CHPAA this concentration of ABA still had a large positive effect (28% stimulation over CHPAA alone, compared to 34% in the control). Thus, it can be concluded that auxin efflux is necessary for the stimulatory effect of low ABA concentrations but that there is no evidence of a role for auxin influx.

Looking at the inhibitory effect of a high ABA concentration, this was surprisingly accentuated in the presence of either of the auxin efflux inhibitors, leading to an 86–89% inhibition of root elongation after 4 days, compared to 48% inhibition with 10 μM ABA alone (**Figure 4B**). By contrast, the auxin influx inhibitor CHPAA had the effect of reducing the inhibitory effect of 10 μM ABA to 6 and 23% of CHPAA alone after 1 and 4 days of treatment respectively (**Figure 4**). These results indicated that auxin influx is important for the root response to high ABA concentrations and that auxin efflux may play a negative role in the mechanism by which high ABA concentrations inhibit root elongation.

**FIGURE 5:**
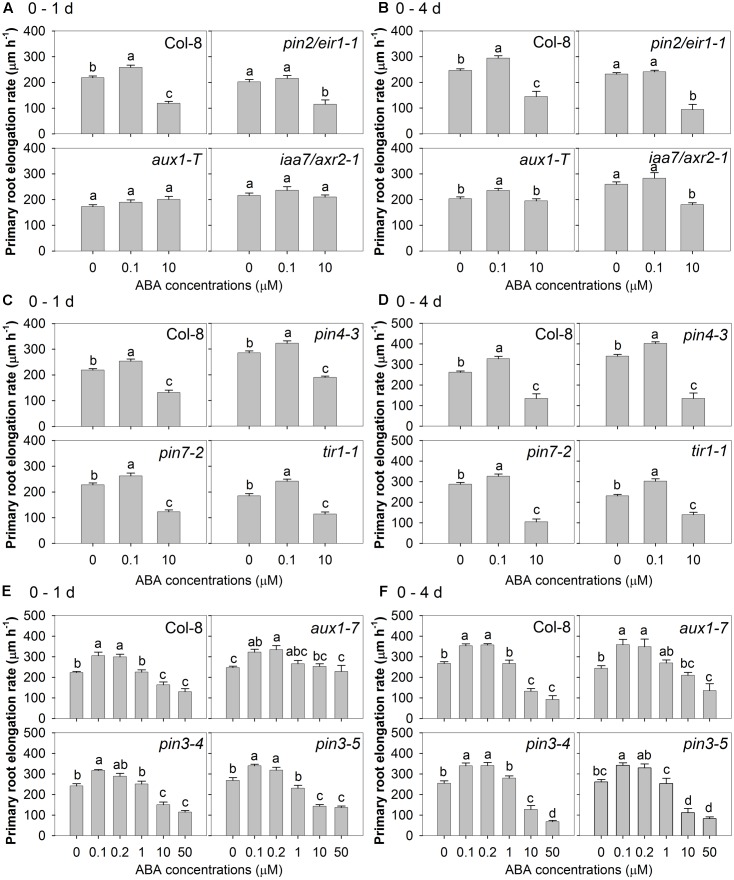
Auxin relevant mutants showed both auxin signalling and auxin transport are required for root growth response to ABA treatments. Primary root elongation rate after the first 24 h treatment of: **(A)** wild-type Col-8, *pin2/eir1-1*, *aux1-T*, *iaa7/axr2-1* (eta^2^ of one-way ANOVA: 0.835, 0.517, 0.118, 0.087); **(C)** Col-8, *pin4-3*, *pin7-2*, *tir1-1* (eta^2^ of one-way ANOVA: 0.798, 0.840, 0.808, 0.798); **(E)** Col-8, *aux1-7*, *pin3-4*, *pin3-5* (eta^2^ of one-way ANOVA: 0.773, 0.419, 0.854, 0.881). Average primary root elongation rate over the 4-day treatment of: **(B)** Col-8, *pin2/eir1-1*, *aux1-T*, *iaa7/axr2-1* (eta^2^ of one-way ANOVA: 0.740, 0.724, 0.324, 0.616); **(D)** Col-8, *pin4-3*, *pin7-2*, *tir1-1* (eta^2^ of one-way ANOVA: 0.790, 0.884, 0.883, 0.828); **(F)** Col-8, *aux1-7*, *pin3-4*, *pin3-5* (eta^2^ of one-way ANOVA: 0.908, 0.705, 0.906, 0.826). **A–F** were results from three experiments separately. In each experiment, seedlings of each line were germinated, chosen and transferred to medium with various ABA concentrations (μM) as described in **Figure 1**. Primary root length was marked after transplanting and the increase of primary root were measured every day. The root elongation rate was calculated for 1 and 4 days after treatments on average. The values are means, and the vertical bars represent standard errors of the means. Data analysed using one-way ANOVA with Tukey’s *post hoc* test and different letters indicate significant differences among ABA treatments in each genotype at *P* < 0.05. Seedling numbers: **(A)** *n* = 12; **(B)** *n* = 6–12; **(C)** *n* = 12; **(D)** *n* = 3–12; **(E)** *n* = 8; **(F)** *n* = 4–8. Similar experiments were done for at least three times with different mutant combinations and similar results were obtrained.

A genetic approach was used to investigate the respective roles of auxin efflux and influx in the root responses to ABA. The allelic auxin influx mutants *aux1-7* and *aux1-T* and five auxin efflux mutants (*pin2/eir1-1*, *pin3-4*, *pin3-5*, *pin4-3*, and *pin7-2*) were treated with a range of concentrations of ABA, and their root elongation rates were compared with that of wild-type after the 1st day and over a 4-day period. The results of three separate experiments are shown in **Figure 5**. Two-way ANOVA was performed for each of those three experiments to test the impact of genotype, ABA treatment and their interaction. In all experiments, irrespective of whether measurements were made in the first 24 h after treatment or 4 days after treatment, there were significant effects of genotype and ABA treatment (*P* < 0.05, Supplementary Table 3). In the first experiment (wild-type, *pin2/eir1-1*, *aux1-T*, and *iaa7/axr2-1*), the results showed that there was significant genotype × ABA treatment interaction effect on the primary root elongation rate in the first 24 h and 4 days after treatment (*P* < 0.0001, Supplementary Table 3). In the second experiment (wild-type, *pin4-3*, *pin7-2*, and *tir1-1*), the interaction between genotype and ABA treatment significantly affected the average primary root elongation rate after 4 days treatment (*P* < 0.0001), but not in the first 24 h after treatment (*P* = 0.42, Supplementary Table 3). In contrast, the results of the third experiment (wild-type, *aux1-7*, *pin3-4*, and *pin 3-5*) suggested that the interaction between genotype and ABA treatment significantly affected the primary root elongation rate in the first 24 h of ABA treatment (*P* < 0.0001), but not the average primary root elongation rate during the 4 days treatment (*P* = 0.11, Supplementary Table 3). Overall, the statistical analysis confirmed that different genotypes responded differently to ABA treatment.

In the first one of these experiments (**Figures 5A,B**), it was found that the *aux1-T* knockout mutant was insensitive to both low and high concentrations of ABA in the 1st day and to the higher concentration of ABA when measured over 4 days, but that a slight positive effect of low concentration of ABA could be detected after 4 days. However, the *aux1-7* missense mutant showed a weaker phenotype, being unaffected in its sensitivity to low [ABA] over either 1 or 4 days (**Figures 5E,F**) and insensitive to high [ABA] during the first 24 h of treatment (**Figure 5E**) but not during the subsequent 3 days (**Figure 5F**). These results are consistent with a role for AUX1-mediated auxin influx in the inhibitory effect of high [ABA], confirming the results obtained with CHPAA (**Figure 4**). An additional role of AUX1 in the stimulatory effect of low [ABA] cannot be ruled out but was only detectable in the early stages of treatment and only in the knockout mutant.

Of the five auxin efflux mutants tested, only *pin2/eir1-1* behaved differently to the wild-type, showing less sensitivity to low [ABA], but normal sensitivity to high [ABA] (**Figures 5A,B**). There was one exception that in one of the three repetitions of this experiment, low [ABA] (0.1 μM) showed similar and weak stimulatory effect in root elongation in *pin2/eir1-1* as in wild-type in the 4 days treatment (by 15% vs. 17% in wild-type, data not shown). However, *pin3-4*, *pin3-5*, *pin4-3*, *pin7-2* all showed similar ABA responses to the wild-type (**Figures 5C–F**). These results are consistent with the evidence from the auxin efflux inhibitors (NPA and TIBA) that blocking auxin efflux did not alleviate the inhibitory effect of high [ABA] (**Figure 4**). It also suggested that the role for auxin efflux in the stimulatory effect of low [ABA] indicated by use of these inhibitors might involve PIN2/EIR1.

Two auxin insensitive mutants (*tir1-1* and *iaa7/axr2-1*) were used to investigate the role of auxin signalling in the root responses to ABA. While the *iaa7/axr2-1* mutant showed reduced sensitivity to both low and high [ABA] (**Figures 5A,B**), the *tir1-1* mutant did not respond differently from the wild-type (**Figures 5C,D**).

## Discussion

### The Positive and Negative Effects of ABA on Root Growth Differ in Their Requirement for Ethylene Signalling

Previous studies identified the importance of ethylene signalling and ethylene biosynthesis for the inhibition of primary root growth by ABA (Ghassemian et al., 2000; Luo et al., 2014). In the present study the objective was to try to understand how different concentrations of exogenous ABA can have opposing effects on root growth and to compare the signalling mechanisms responsible for the positive and negative responses. Use of the ethylene perception inhibitor STS (**Figures 2C,D**) along with three ethylene-insensitive mutants (*etr1-1, ein2-1*, and *ein3-1*) (**Figure 3**) confirmed that ethylene signalling was important for the inhibitory effect of high [ABA] under our experimental conditions.

In this study, the ability of the ethylene biosynthesis inhibitor AVG to completely suppress the inhibitory effect of high [ABA] (**Figures 2A,B**) is consistent with recent evidence that ABA inhibits root growth in Arabidopsis by promoting ethylene biosynthesis (Luo et al., 2014). The discrepancy between the present results and an earlier finding (Ghassemian et al., 2000) that AVG did not overcome the inhibitory effect of high [ABA], and even increased the degree of inhibition, could be attributable to the earlier authors’ use of higher concentrations of AVG than those used here. These higher concentrations may themselves have been inhibitory to root growth through AVG’s reported effects on auxin biosynthesis (Soeno et al., 2010). A recent report by Rowe et al. (2016) suggested that severe osmotic stress (-1.2 MPa) inhibited Arabidopsis root growth independently of either ABA or ethylene. However, in their experimental system, they did not observe the promotion of root growth by either low exogenous ABA or moderate osmotic stress (-0.37 MPa) that have been reported here and in other systems (Creelman et al., 1990; Xu et al., 2013; Rowe et al., 2016). Some other studies have also suggested that ABA and ethylene can antagonise each other and that ethylene can prevent ABA accumulation or modulate cellular sensitivity to ABA under stress (Wilkinson and Davies, 2010; Wilkinson et al., 2012; Chen et al., 2013). Therefore, the interaction between ABA and ethylene in stressed plants can be complex.

In contrast to the ethylene-dependence of the inhibitory effect of high [ABA], there was no evidence of any involvement of ethylene biosynthesis or signalling in the stimulatory effect of low [ABA]: neither AVG nor STS blocked the stimulatory effect (**Figure 2**) and the ethylene-insensitive mutants still responded positively to low [ABA] (**Figure 3**). These results indicate that the opposing effects of high and low ABA concentrations on root elongation operate through different signalling pathways.

Among the three ethylene-insensitive mutants tested here, the order of the effect on the root response to high [ABA] is *ein2-1* > *etr1-1* > *ein3-1*. These results were consistent with the report that *ein2-1* has stronger impairment in ethylene responsiveness than *etr1-1* does (van Loon et al., 2006). In addition, the mutant alleles of *ein3* were previously reported to be less insensitive to ethylene than the strong alleles of *etr1* and *ein2* (Roman et al., 1995; Chao et al., 1997). Six members of the *EIN3* family have been identified, with *EIL1* being one the most closely related to *EIN3* (Alonso et al., 2003b). A completely ethylene-insensitive phenotype has been reported in an *ein3 eil1* double mutant, while the *ein3* and *eil1* single mutants have incomplete ethylene insensitivity (Alonso et al., 2003a,b). This indicates that there is partial redundancy of function between *EIN3* and *EIL1*, which would provide an explanation for the weaker phenotype of the *ein3-1* mutant in our experiments.

### Auxin Signalling Is Involved in Both the Positive and Negative Responses to Exogenous ABA

A role for auxin in the inhibitory effect of high [ABA] on Arabidopsis root growth has already been established from a number of studies using mutants defective in auxin transport and signalling (Belin et al., 2009; Wang et al., 2011; Thole et al., 2014; Zhao et al., 2015). Additionally, the distribution of GFP in the root tip of the *DR5::GFP* auxin reporter line was altered by adding either low or high [ABA] compared with the control (Supplementary Figure 2), which is consistent with the idea that auxin is involved in regulating root growth responses to both low and high [ABA]. However, similar distribution of GFP was observed for both low and high [ABA] treatments. These results suggest that auxin may not involve in root growth responses to low and high [ABA] through changing auxin maximum pattern in root tip but other means, e.g., plant sensitivity to auxin. In addition, the GFP expression patterns observed in this study were 3 days after the start of treatment, so we cannot rule out the possibility that there were short-term differences in the effects of the high and low [ABA] treatments on *DR5::GFP* expression that were missed in these experiments.

To look further into the involvement of auxin signalling in the root responses to both low and high [ABA], two mutants defective in components of the auxin signalling were used. The finding that *iaa7/axr2-1* had reduced sensitivity to both the inhibitory effect of 10 μM ABA and the stimulatory effect of 0.1 μM ABA (**Figures 5A,B**) indicates that both the low and high [ABA] responses of root growth depend on auxin signalling. It has previously been shown that ABA represses the expression of the *IAA7/AXR2* gene independent of auxin, leading to the suggestion that *IAA7/AXR2* is at the nexus of crosstalk between ABA and auxin signalling pathways by acting as a negative regulator of both pathways (Belin et al., 2009). On the other hand, the gain-of-function mutant *iaa7/axr2-1* has an amino acid substitution in the conserved domain II of IAA7/AXR2, which results in the accumulation of this AUX/IAA family protein and leads to repression of a series of downstream auxin responses (Nagpal et al., 2000; Gray et al., 2001). Thus, it may be that downstream components of the auxin signalling pathway, rather than IAA7/AXR2 *per se*, are required for both low and high [ABA] effects on root growth. The lack of a similar phenotype in another auxin signalling mutant *tir1-1* (**Figures 5C,D**) is consistent with an earlier report that the *tir1-1* mutant showed normal repression of embryonic axis elongation in response to ABA (Belin et al., 2009). This could indicate that either other F-box proteins are involved or it could be explained by genetic redundancy amongst members of this small family of auxin receptors (Dharmasiri et al., 2005; Parry et al., 2009).

### Differences between the Positive and Negative Responses to ABA in Their Requirements for Auxin Influx and Efflux

Two previous studies reported that *aux1* auxin influx mutants were less sensitive to high concentrations of ABA than wild-type (Belin et al., 2009; Thole et al., 2014) and a *pin2* auxin efflux mutant was also found to be insensitive to ABA-dependent repression of both hypocotyl and radicle elongation (Belin et al., 2009). The insensitive phenotype with respect to high [ABA] in *aux1* was confirmed in the present study (**Figures 5A,B,E,F**) and the role for auxin influx in ABA’s inhibitory effect on root growth was further supported by the ability of the auxin influx inhibitor CHPAA to antagonise this response to high [ABA] (**Figure 4**). Previously, Rowe et al. (2016) found that root growth in the *aux1*-7 mutant responded similarly to the wild-type under moderate and severe osmotic stresses. However, they collected root length data only after 4 days of osmotic stress (between 5 and 9 days after germination), which would not necessarily reveal the very early ABA response of *aux1-7* as shown in **Figures 5E,F**.

How *aux1* mutations affect the stimulatory effect of low [ABA] in our study was less clear-cut: an absence of stimulation of root growth by low [ABA] was only observed in the *aux1-T* knockout mutant during the first 24 h of treatment (**Figures 5A,B**). The wild-type phenotype was seen in the *aux1-7* missense mutant with low [ABA] treatments (**Figures 5E,F**). Nevertheless, CHPAA failed to block the stimulatory effect of low [ABA] (**Figure 4**), indicating that there are differences between the positive and negative responses to ABA in their requirement for auxin influx.

When the positive and negative responses to ABA were compared for their requirement for auxin efflux, a distinct difference was found. No evidence of a positive role for auxin efflux in the inhibitory effect of ABA was obtained, based on the phenotypes of the *pin2/eir1-1, pin3-4, pin3-5, pin4-3*, and *pin7-2* mutants (**Figures 5A-F**) and the inability of two auxin efflux inhibitors (NPA and TIBA) to overcome the inhibitory effect (**Figure 4**). On the other hand, the enhanced degree of inhibition by 10 μM ABA that was seen in the presence of either NPA or TIBA in the latter experiment suggests that auxin efflux may play a role in counteracting the inhibitory effect of high [ABA]. PIN1 is an important auxin efflux carrier that regulates polar auxin transport and supports auxin transport from shoot to root (Gälweiler et al., 1998; Růžička et al., 2007). Reduced *PIN1* expression can normally cause auxin deficiency in roots, which is accompanied by growth inhibition (Blilou et al., 2005; Fernández-Marcos et al., 2011). Application of high [ABA] (10 μM) to well-watered plants reduced *PIN1* expression in the root tip (Rowe et al., 2016). Thus, the application of an auxin efflux inhibitor (NPA or TIBA) in combination with the high [ABA] treatment under well-watered conditions is likely to result in even lower auxin levels in root tips and much stronger inhibition of root growth. By contrast, both NPA and TIBA were successful in blocking the stimulatory effect of low [ABA] and the *pin2/eir1-1* mutant (but not the other tested *pin* mutants) was also defective in its response to low [ABA] (**Figures 4**, **5A,B**). This evidence of the importance of auxin efflux in the response to low [ABA] agrees with a previous report that TIBA was able to partially suppress the positive effect of a low concentration of ABA on root growth in rice (Zhao et al., 2015).

Of the four *PIN* genes of which the role in ABA responses was tested (*PIN2, PIN3, PIN4*, and *PIN7*), it is notable that *PIN2* is the only one that is expressed in the lateral root cap (Blilou et al., 2005; Kleine-Vehn et al., 2010; Band et al., 2014). The *pin2/eir1-1* mutant also shows an altered pattern of distribution of the auxin maximum in the root tip compared to other *pin* mutants (Ottenschläger et al., 2003; Blilou et al., 2005). It is possible that the reduced sensitivity to low [ABA] that is seen in the *pin2/eir1-1* mutant might be related to specific alterations in auxin distribution that arise from loss of PIN2/EIR1’s contribution to auxin efflux in the lateral root cap. On the other hand, auxin influx and efflux carriers act redundantly and a single mutation may have limited effect to ABA treatments. For example, PIN3 in the columella cells is proved to be important for gravity-sensing, but *pin3* mutant showed marginal defect in gravity response, which is related to the redundancy of *PIN3*, *PIN4*, and *PIN7* (Blilou et al., 2005; Kleine-Vehn et al., 2010).

**FIGURE 6:**
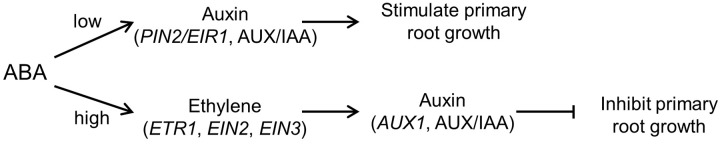
A model for the involvement of ethylene and auxin in root growth responses to different ABA treatments. In the model, ABA regulates root growth through two distinct pathways: (1) an ethylene-independent stimulatory pathway that operates at low [ABA] and requires auxin signalling and auxin efflux through PIN2/EIR1; and (2) an ethylene-dependent inhibitory pathway that operates at high [ABA] and that also requires auxin signalling and auxin influx through AUX1. Ethylene regulates root growth through downstream auxin is based on the report that *aux1-T* mutant exhibited ACC-resistant root growth (Růžička et al., 2007).

The results in this study provide evidence that the stimulatory effect of low ABA concentrations on root growth operates through an ethylene-independent pathway, and requires auxin signalling and auxin transport by the PIN2/EIR1 auxin efflux carrier (**Figure 6**). However, the inhibitory effect seen at high ABA concentrations is through an ethylene-dependent pathway that requires auxin signalling and auxin influx through AUX1 (**Figure 6**). Růžička et al. (2007) found ethylene inhibits root elongation by stimulating auxin biosynthesis and auxin transport toward the root elongation zone. Ethylene failed to activate auxin response and inhibit root growth in mutants that are defective in auxin perception or basipetal auxin transport (i.e., *tir1-1*, *aux1-T*) (Růžička et al., 2007). In future it will be important to test the hypothesis that the same distinct pathways also account for the analogous biphasic response of root growth to different degrees of water deficit (Sharp and Davies, 1979; van der Weele et al., 2000; Xu et al., 2013). The resulting insight into how root growth responds to different degrees of soil drying could have agronomic significance in helping to manage crop development and to develop crop varieties whose root responses to soil drying are more beneficial to crop productivity in a changing climate and an increasingly water-scarce world.

## Author Contributions

XL, LC, BF, and WD designed all of the experiments. XL performed all the lab work and data analysis. XL, LC, BF, and WD wrote the manuscript.

## Conflict of Interest Statement

The authors declare that the research was conducted in the absence of any commercial or financial relationships that could be construed as a potential conflict of interest.

## Abbreviations

ABA: abscisic acid
[ABA]: abscisic acid concentration
ACC: 1-aminocyclopropane-1-carboxylic acid
Ag^+^: silver ion
ARF: auxin response factor
AUX/IAA: auxin/indole-3-acetic acid
AUX1: AUXIN 1 (auxin influx transporter)
AVG: aminoethoxyvinylglycine (ethylene biosynthesis inhibitor)
CHPAA: 3-chloro-4-hydroxyphenylacetic acid (auxin influx inhibitor)
GFP: green fluorescent protein
NPA: *N*-1-naphthylphthalamidic acid (auxin efflux inhibitor)
PIN: PIN-FORMED (auxin efflux transporter)
STS: silver thiosulfate (ethylene signalling inhibitor)
TIBA: 2,3,5-triiodobenzoic acid (auxin efflux inhibitor).
