# Supplementary material for: The Biphasic Root Growth Response to Abscisic Acid in Arabidopsis Involves Interaction with Ethylene and Auxin Signalling Pathways
Source: Front Plant Sci. 2017 Aug 25;8:1493. doi: 10.3389/fpls.2017.01493 (PMC5574904; doi:10.3389/fpls.2017.01493)
Supplement: Supplementary file 1 [file Data_Sheet_1.pdf]

Supplementary Material

The biphasic root growth response to abscisic acid in Arabidopsis involves interaction with ethylene and auxin signalling pathways

Xiaoqing Li[1, *, †], Lin Chen[1, *, ‡], Brian G. Forde[1], William J. Davies[1]

1 Lancaster Environment Centre, Lancaster University, Bailrigg, Lancaster, LA1 4YQ, UK

*** Correspondence:** Lin Chen lin.chen@york.ac.uk; yncllily@hotmail.com; Xiaoqing Li lixqymail@gmail.com

† Present address: CSIRO Agriculture and Food, GPO Box 1700, Canberra, ACT 2601, Australia

‡ Present address: Department of Biology, University of York, York, YO10 5DD, UK

Supplementary Table 1. Primary root length in the beginning and the end of each experiment. The values are means ± standard errors. Data analysed by using one-way ANOVA with Tukey's *post hoc* test at the $P < 0.05$ level. Different letters indicate significant differences among treatments in each genotype of every experiment either at the beginning or the end of that experiment.

			**Primary root length (mm)**	
**Figure**	**Genotype**	**Treatment**	**Beginning**	**End**
Figure 1	Wild-type	Control	11.4 ± 0.3 a	43.2 ± 1.2 b
		0.1 µM ABA	10.8 ± 0.3 ab	51.0 ± 1.0 a
		1 µM ABA	10.5 ± 0.2 ab	38.3 ± 1.8 b
		10 µM ABA	9.8 ± 0.4 b	26.5 ± 1.7 c
Figure 2A & B	Wild-type	Control	11.4 ± 0.3 ab	33.1 ± 1.0 cd
		0.3 µM AVG	11.0 ± 0.2 ab	35.7 ± 0.5 bcd
		0.5 µM AVG	11.9 ± 0.4 a	34.0 ± 1.3 cd
		0.1 µM ABA	12.1 ± 0.4 a	39.8 ± 1.6 ab
		0.1 µM ABA + 0.3 µM AVG	11.3 ± 0.3 ab	41.4 ± 0.6 a
		0.1 µM ABA + 0.5 µM AVG	11.4 ± 0.2 ab	37.6 ± 1.2 abc
		10 µM ABA	11.8 ± 0.3 ab	22.0 ± 0.9 e
		10 µM ABA + 0.3 µM AVG	10.5 ± 0.3 b	30.3 ± 1.8 d
		10 µM ABA + 0.5 µM AVG	11.2 ± 0.3 ab	30.2 ± 2.3 d
Figure 2C & D	Wild-type	Control	10.3 ± 0.4 a	40.8 ± 1.0 b
		10 µM STS	10.6 ± 0.3 a	40.3 ± 1.3 b
		0.1 µM ABA	10.2 ± 0.4 a	48.5 ± 0.9 a
		0.1 µM ABA + 10 µM STS	11.3 ± 0.5 a	48.8 ± 1.3 a
		10 µM ABA	10.3 ± 0.4 a	26.7 ± 1.4 c
		10 µM ABA + 10 µM STS	10.5 ± 0.6 a	28.1 ± 1.3 c
Figure 3	Wild-type	Control	12.9 ± 0.2 a	36.1 ± 0.4 b
		0.1 µM ABA	12.7 ± 0.2 a	43.1 ± 0.6 a
		0.2 µM ABA	12.7 ± 0.2 a	42.9 ± 0.7 a
		1 µM ABA	12.5 ± 0.2 a	35.9 ± 0.9 b
		5 µM ABA	12.2 ± 0.2 a	26.3 ± 1.1 c
		10 µM ABA	12.2 ± 0.2 a	24.7 ± 0.8 cd
		30 µM ABA	12.6 ± 0.2 a	22.1 ± 0.6 d
Figure 3	*etr1-1*	Control	10.3 ± 0.7 a	34.8 ± 1.3 ab
		0.1 µM ABA	10.0 ± 0.8 a	39.6 ± 1.3 a
		0.2 µM ABA	9.8 ± 0.7 a	38.7 ± 1.2 a
		1 µM ABA	10.1 ± 0.7 a	33.4 ± 1.2 bc
		5 µM ABA	9.6 ± 0.8 a	28.3 ± 1.1 d
		10 µM ABA	9.9 ± 0.7 a	28.7 ± 0.9 cd
		30 µM ABA	9.3 ± 0.7 a	26.1 ± 1.1 d
	ein2-1	Control	12.9 ± 0.6 a	45.4 ± 0.8b
		0.1 µM ABA	13.0 ± 0.6 a	49.8 ± 0.7 a
		0.2 µM ABA	13.3 ± 0.7 a	49.6 ± 1.0 a
		1 µM ABA	13.1 ± 0.6 a	46.9 ± 1.0 ab
		5 µM ABA	12.9 ± 0.6 a	40.2 ± 0.9 c
		10 µM ABA	13.2 ± 0.6 a	39.4 ± 0.8 c
		30 µM ABA	13.2 ± 0.7 a	37.1 ± 1.4 c
	ein3-1	Control	11.3 ± 0.2 a	35.0 ± 0.4 c
		0.1 µM ABA	11.8 ± 0.3 a	44.8 ± 0.8 a
		0.2 µM ABA	11.3 ± 0.2 a	42.9 ± 0.5 a
		1 µM ABA	11.5 ± 0.2 a	38.2 ± 0.8 b
		5 µM ABA	11.7 ± 0.2 a	29.3 ± 0.8 d
		10 µM ABA	11.5 ± 0.2 a	26.8 ± 0.7 de
		30 µM ABA	11.5 ± 0.2 a	24.7 ± 0.7 e
Figure 4	Wild-type	0.1% DMSO (Control)	10.5 ± 0.2 a	32.6 ± 0.6 b
		10 µM NPA	10.2 ± 0.2 a	24.7 ± 0.6 cde
		10 µM TIBA	10.9 ± 0.3 a	27.1 ± 0.6 c
		10 µM CHPAA	10.5 ± 0.2 a	26.0 ± 0.5 cd
		0.1 µM ABA + 0.1% DMSO	10.4 ± 0.2 a	40.0 ± 0.6 a
		0.1 µM ABA + 10 µM NPA	10.8 ± 0.3 a	24.8 ± 2.6 cde
		0.1 µM ABA + 10 µM TIBA	10.3 ± 0.2 a	26.7 ± 1.1 c
		0.1 µM ABA + 10 µM CHPAA	11.1 ± 0.2 a	31.0 ± 1.3 b

Supplementary Table 1 (continued)

Figure	Genotype	Treatment	Primary root length (mm) Beginning	End
Figure 4	Wild-type	10 µM ABA + 0.1% DMSO	10.8 ± 0.2 a	22.0 ± 0.4 e
		10 µM ABA + 10 µM NPA	10.4 ± 0.2 a	12.0 ± 0.2 f
		10 µM ABA + 10 µM TIBA	10.2 ± 0.3 a	12.6 ± 0.5 f
		10 µM ABA + 10 µM CHPAA	10.6 ± 0.2 a	22.3 ± 0.8 de
Figure 5				
A & B	Wild-type	Control	10.4 ± 0.3 a	33.5 ± 0.7 b
		0.1 µM ABA	10.2 ± 0.3 a	37.9 ± 1.0 a
		10 µM ABA	10.7 ± 0.3 a	24.3 ± 2.1 c
	pin2/eir1-1	Control	11.3 ± 0.4 a	33.1 ± 0.6 a
		0.1 µM ABA	10.7 ± 0.4 a	33.4 ± 0.8 a
		10 µM ABA	11.1 ± 0.4 a	20.0 ± 1.9 b
	aux1-T	Control	10.5 ± 0.4 a	29.5 ± 1.0 ab
		0.1 µM ABA	10.0 ± 0.3 a	32.0 ± 0.9 a
		10 µM ABA	10.1 ± 0.4 a	28.4 ± 0.9 b
	iaa7/axr2-1	Control	11.3 ± 0.7 a	35.7 ± 1.4 a
		0.1 µM ABA	11.3 ± 0.6 a	38.1 ± 2.9 a
		10 µM ABA	12.4 ± 0.4 a	29.1 ± 0.7 b
C & D	Wild-type	Control	10.5 ± 0.1 a	35.1 ± 0.6 b
		0.1 µM ABA	10.5 ± 0.2 a	41.2 ± 1.3 a
		10 µM ABA	10.3 ± 0.2 a	23.1 ± 2.0 c
	pin4-3	Control	13.7 ± 0.2 a	45.6 ± 0.8 b
		0.1 µM ABA	13.8 ± 0.3 a	51.4 ± 0.9 a
		10 µM ABA	14.0 ± 0.4 a	25.9 ± 3.4 c
	pin7-2	Control	12.1 ± 0.1 a	39.0 ± 0.8 b
		0.1 µM ABA	12.2 ± 0.2 a	42.8 ± 1.0 a
		10 µM ABA	12.4 ± 0.3 a	21.5 ± 1.3 c
	tir1-1	Control	11.4 ± 0.1 a	33.1 ± 0.7 b
C & D	*tir1-1*	0.1 µM ABA	11.4 ± 0.2 a	39.3 ± 1.1 a
		10 µM ABA	11.4 ± 0.3 a	24.3 ± 1.3 c
E & F	Wild-type	Control	14.0 ± 0.4 a	39.7 ± 0.9 b
		0.1 µM ABA	13.8 ± 0.6 a	47.4 ± 1.1 a
		0.2 µM ABA	13.8 ± 0.5 a	48.0 ± 1.0 a
		1 µM ABA	13.2 ± 0.6 a	38.8 ± 2.1 b
		10 µM ABA	13.6 ± 0.7 a	26.3 ± 1.3 c
		50 µM ABA	14.3 ± 0.9 a	23.6 ± 2.4 c
	aux1-7	Control	11.4 ± 0.3 a	34.8 ± 1.4 bc
		0.1 µM ABA	11.6 ± 0.4 a	45.8 ± 3.1 a
		0.2 µM ABA	12.0 ± 0.4 a	45.5 ± 4.0 ab
		1 µM ABA	11.3 ± 0.6 a	36.8 ± 1.8 abc
		10 µM ABA	12.1 ± 0.5 a	32.2 ± 1.4 c
		50 µM ABA	11.2 ± 0.5 a	17.4 ± 0.8 d
	pin3-4	Control	12.8 ± 0.4 a	37.2 ± 0.9 b
		0.1 µM ABA	12.4 ± 0.2 a	44.9 ± 1.4 a
		0.2 µM ABA	11.6 ± 0.4 a	44.3 ± 1.5 a
		1 µM ABA	11.1 ± 0.4 a	37.9 ± 1.1 b
		10 µM ABA	11.6 ± 0.9 a	24.0 ± 2.2 c
		50 µM ABA	10.9 ± 0.4 a	17.4 ± 0.6 d
	pin3-5	Control	13.7 ± 0.2 a	39.4 ± 1.1 ab
		0.1 µM ABA	12.8 ± 0.3 a	45.3 ± 1.3 a
		0.2 µM ABA	12.8 ± 0.5 a	44.4 ± 2.1 ab
		1 µM ABA	12.7 ± 0.4 a	37.2 ± 2.7 b
		10 µM ABA	13.2 ± 0.4 a	23.9 ± 2.2 c
		50 µM ABA	12.2 ± 0.4 a	20.0 ± 0.8 c
Supplementary Figure 1	*DR5::GFP*	Control	17.0 ± 0.4 a	30.4 ± 0.7 b
		0.1 µM ABA	17.2 ± 0.5 a	38.5 ± 1.0 a
		10 µM ABA	17.3 ± 0.9 a	21.5 ± 1.0 c

Supplementary Table 2. Impact of genotype (wild-type, *etr1-1*, *ein2-1* and *ein3-1*) and ABA treatment on primary root elongation rate. (A) Absolute values of primary root elongation rate. (B) Relative primary root elongation rate (in each genotype, the mean root elongation rate of plants without ABA treatment is set as 1). Data analysed by using two-way ANOVA with genotype and treatment as main factors. Degrees of freedom (df), sums of squares (SS), *F* values, *P* values, eta^2 and partial eta^2 from ANOVA are presented. Significance: *, 0.05; **, 0.001; ***, 0.0001.

	**Primary root elongation rate**											
	**0−24 h**						**0−4 d**					
	**SS**	**df**	***F* value**	***P* value**	**Eta^2**	**Partial eta^2**	**SS**	**df**	***F* value**	***P* value**	**Eta^2**	**Partial eta^2**
**A**												
Absolute values of primary root elongation rate												
Genotype	3154476.8	3	628.6	***	0.505	0.714	1652142.1	3	409.5	***	0.284	0.633
Treatment	1485213.6	6	148.0	***	0.238	0.540	3024066.2	6	374.7	***	0.520	0.760
Genotype × treatment	338018.3	18	11.2	***	0.054	0.211	181012.9	18	7.5	***	0.031	0.159
Residuals	1264605.6	756					956263.5	711				
**B**												
Relative primary root elongation rate (the root elongation rate of plants without ABA treatment is set as 1 for each genotype)												
Genotype	7.7	3	94.5	***	0.123	0.273	0.9	3	15.4	***	0.014	0.061
Treatment	27.2	6	167.6	***	0.435	0.571	43.8	6	382.7	***	0.693	0.764
Genotype × treatment	7.2	18	14.7	***	0.115	0.260	4.9	18	14.3	***	0.078	0.266
Residuals	20.4	756					13.6	711				

Supplementary Table 3. Impact of genotype and ABA treatment on primary root elongation rate. Data analysed by using two-way ANOVA with genotype and treatment as main factors. Degrees of freedom (df), sums of squares (SS), *F* values, *P* values, eta^2 and partial eta^2 from ANOVA are presented. Significance: *, 0.05; **, 0.001; ***, 0.0001.

	**Primary root elongation rate**											
	**0−24 h**						**0−4 d**					
	**SS**	**df**	***F* value**	***P* value**	**Eta^2**	**Partial eta^2**	**SS**	**df**	***F* value**	***P* value**	**Eta^2**	**Partial eta^2**
**A**												
Genotypes are wild-type, *pin2/eir1-1* , *aux1-T* and *iaa7/axr2-1* ; treatments are 0, 0.1 and 10 µM ABA												
Genotype	36369.6	3	9.1	***	0.087	0.171	43767.2	3	11.3	***	0.091	0.233
Treatment	100398.8	2	37.7	***	0.241	0.363	234130.6	2	90.3	***	0.486	0.619
Genotype × treatment	104481.0	6	13.1	***	0.250	0.373	60270.5	6	7.7	***	0.125	0.295
Residuals	175928.4	132					143934.8	111				
**B**												
Genotypes are wild-type, *pin4-3* , *pin7-2* and *tir1-1* ; treatments are 0, 0.1 and 10 µM ABA												
Genotype	146644.6	3	63.3	***	0.215	0.590	53389.0	3	15.8	***	0.064	0.312
Treatment	428337.3	2	277.3	***	0.628	0.808	637138.3	2	282.0	***	0.758	0.844
Genotype × treatment	4695.1	6	1.0	0.42	0.007	0.044	32301.4	6	4.8	***	0.038	0.216
Residuals	101945.5	132					117485.4	104				
**C**												
Genotypes are wild-type, *aux1-7* , *pin3-4* and *pin3-5* ; treatments are 0, 0.1, 0.2, 1, 10 and 50 µM ABA												
Genotype	79017.2	3	18.1	***	0.069	0.244	20080.4	3	3.9	*	0.012	0.075
Treatment	738930.0	5	101.7	***	0.647	0.752	1320216.0	5	153.1	***	0.811	0.842
Genotype × treatment	79765.9	15	3.7	***	0.070	0.246	38902.4	15	1.5	0.11	0.024	0.135
Residuals	244173.2	168					248275.3	144				

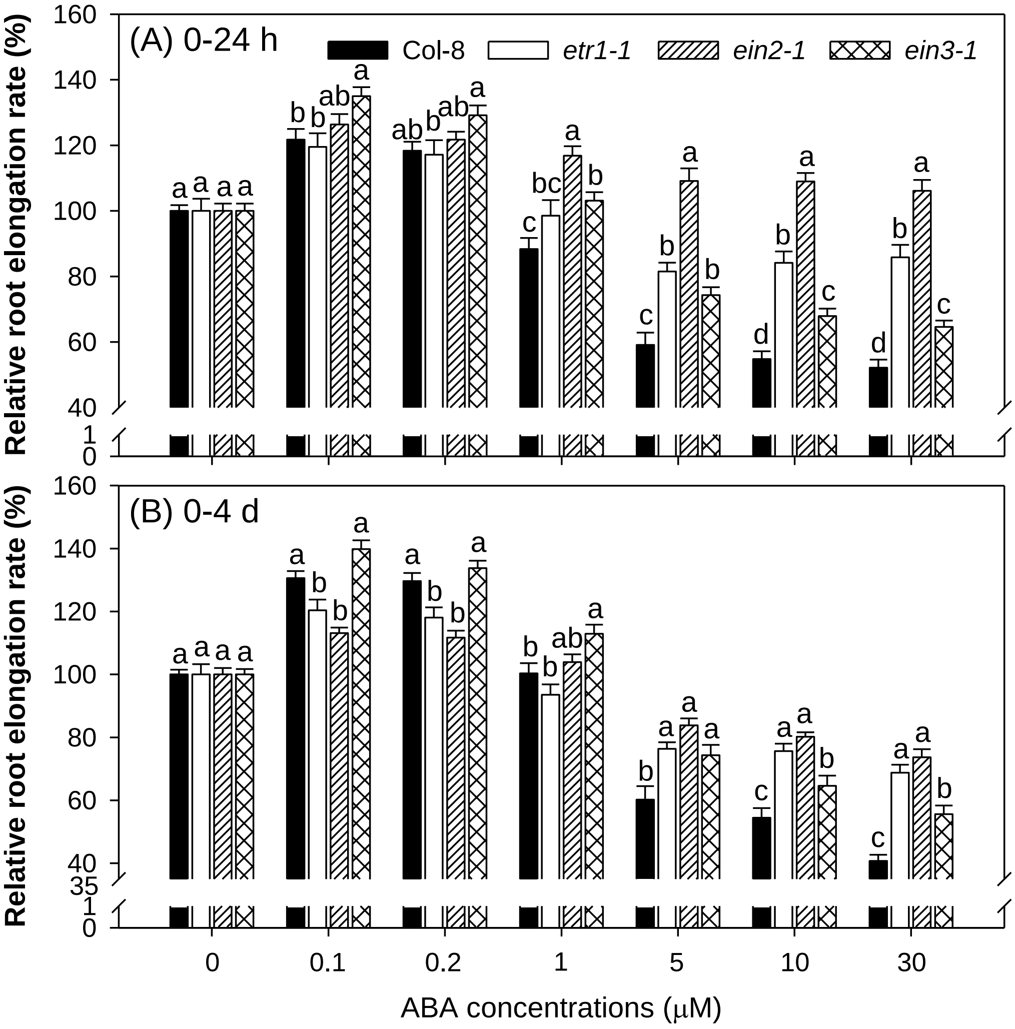

Supplementary Figure 1. Relative primary root elongation rate of four genotypes (wild-type Col-8, *etr1-1*, *ein2-1* and *ein3-1*) under seven ABA treatments (in each genotype, the mean root elongation rate of plants without ABA treatment is set as 1). (A) 0–24 h, (B) 0–4 d. The values are means, and the vertical bars represent standard errors of the means. Data analysed using one-way ANOVA with Tukey's *post hoc* test (n = 21–28) and different letters indicate significant differences among different genotypes under the same ABA treatment at $P < 0.05$. Eta^2 of one-way ANOVA for 0, 0.1, 0.2, 1, 5, 10 and 30 μM ABA treatments are (A) 0, 0.103, 0.071, 0.241, 0.537, 0.672 and 0.639 respectively, and (B) 0, 0.358, 0.298, 0.174, 0.225, 0.364 and 0.505 respectively.

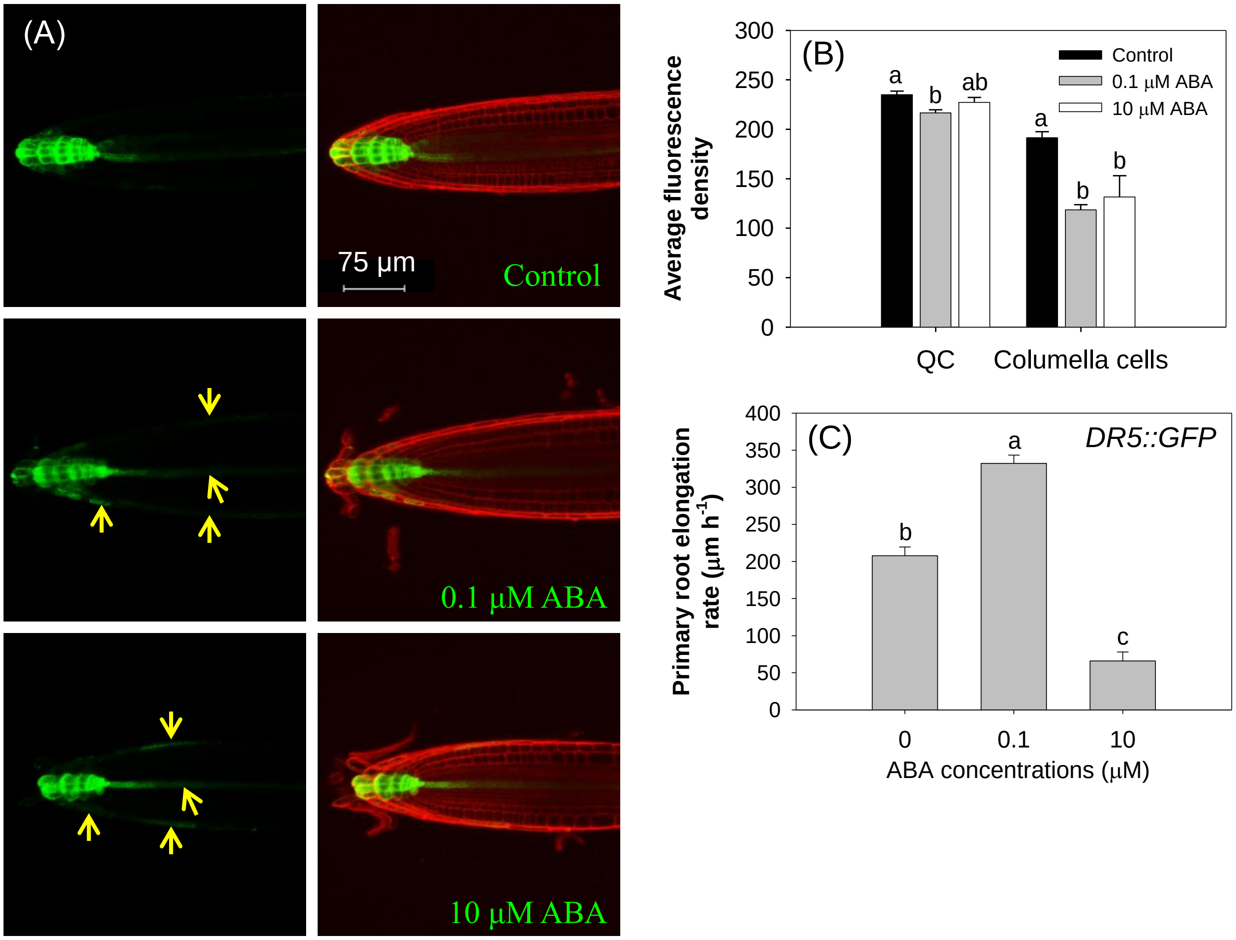

Supplementary Figure 2. ABA treatments induced GFP signal redistribution in root tips of *DR5::GFP* line. (A) Merged z-stack images of root tips (3-day after ABA treatments). (B) Average GFP fluorescence density in quiescent centre (QC) and columella cells (per unit area). (C) Primary root elongation rate of *DR5::GFP* line during the 3-day ABA treatment (n = 5–6). Col-8 seedlings were germinated, chosen and transferred to medium with various ABA concentrations (µM) as described in Figure 1. There were six seedlings per plate and three of them were chosen for imaging. Confocal images were merged from seven image sections. The interval was 1.1965 µm between every two sequential image sections. The values are means, and the vertical bars represent standard errors of the means. Data analysed using one-way ANOVA with Tukey's *post hoc* test and different letters indicate significant differences among ABA treatments at $P < 0.05$ in (B) QC (eta^2: 0.642) or columella cells (eta^2: 0.743); (C) primary root elongation rate (eta^2: 0.952). Arrows point out where a changed pattern of GFP signal can be seen in ABA treated root (the lateral root cap and the middle of vascular tissue).
